# Supplementary material for: A Systematic Review of Mechanisms Underpinning Psychological Change Following Nature Exposure in an Adolescent Population
Source: Int J Environ Res Public Health. 2022 Oct 3;19(19):12649. doi: 10.3390/ijerph191912649 (PMC9566701; doi:10.3390/ijerph191912649)
Supplement: Supplementary file 1 [file ijerph-19-12649-s001.zip › ijerph-1916671-supplementary.pdf]

Supplementary Material

File S1. Electronic Search Strategies for Each Database: SCOPUS, Web of Science and PsychINFO
SCOPUS

(TITLE-ABS-KEY ("contact with nature" OR "nature exposure" OR "exposure to nature" OR "natur* experience*" OR "access to nature" OR "green space*" OR greenspace OR greenery OR greenness OR forests OR "forest school" OR "shinrin yoku" OR "shinrin-yoku" OR "forest bathing" OR "forest environment" OR wilderness OR "blue space" OR park OR parks OR woodlands OR countryside OR "urban forest" OR "outdoor adventure interventions" OR "adventure therapy" OR gardening OR "natur* environment*" OR "outdoor adventure education" OR "adventure education" OR "adventure program" OR outdoors OR "green exercise" OR "nature therap*" OR "green play" OR "nature therap*" OR ecotherapy* OR "school landscape")) AND (TITLE-ABS-KEY ("mental health" OR anxiety OR depress* OR mood OR well-being OR wellbeing OR well-being OR "strengths and difficulties")) AND (TITLE-ABS-KEY (adolescen* OR teen*or "young people" OR "young adult*" OR youth*))

Web of Science

#4: #3 AND #2 AND #1
#3: TS =(adolescen* OR teen*OR "young people" OR "young adult*" OR youth*)
#2: TS=("mental health" OR anxiety OR depress* OR mood OR well-being OR wellbeing OR well-being OR "strengths and difficulties")
#1: TS
=("contact with nature" OR "nature exposure" OR "exposure to nature" OR "natur* experience*" OR "a ccess to nature" OR "green space*" OR greenspace OR greenery OR greenness OR forests OR "forest sc hool" OR "shinrin yoku" OR "shinrin-yoku" OR "forest bathing" OR "forest environment" OR wilderness OR "blue space" OR park OR park s OR woodlands OR countryside OR "urban forest" OR "outdoor adventure interventions" OR "adven ture therapy" OR gardening OR "natur* environment*" OR "outdoor adventure education" OR "adven ture education" OR "adventure program" OR outdoors OR "green exercise" OR "nature therap*" OR "g reen play" OR "nature therap*" OR ecotherapy* OR "school landscape")

PsychINFO

1:("contact with nature" OR "nature exposure" OR "exposure to nature" OR "natur* experience*" OR "a ccess to nature" OR "green space*" OR greenspace OR greenery OR greenness OR forests OR "forest sc hool" OR "shinrin yoku" OR "shinrin-yoku" OR "forest bathing" OR "forest environment" OR wilderness OR "blue space" OR park OR park s OR woodlands OR countryside OR "urban forest" OR "outdoor adventure interventions" OR "adven ture therapy" OR gardening OR "natur* environment*" OR "outdoor adventure education" OR "adven ture education" OR "adventure program" OR outdoors OR "green exercise" OR "nature therap*" OR "g reen play" OR "nature therap*" OR ecotherapy* OR "school landscape")
2: ("mental health" or anxiety or depress* or mood or well-being or wellbeing or well-being or "strengths and difficulties").tw.
3. (adolescen* or teen* or "young people" or "young adult*" or youth*).tw.
4. 1 and 2 and 3

Table S1. Summary of the Measurement Tools Used in Each Study

Outcome	Tool or measurement	Studies
Mood and Affect	Profile of Mood States	Bielinis et al. (2019); Bielinis et al. (2021); Bielinis, Omelan et al. (2018); Bielinis, Takayama et al. (2018); Mao et al. (2012); Park et al. (2011); Park et al. (2010).
	Profile of Mood States – Short Form	Lee et al. (2011); Lee et al. (2014); Song et al. (2014); Song et al. (2015); Song et al . (2013); Takayama et al. (2011); Tsunetsugu et al. (2013).
	Profile of Mood States – Adolescents	Wood et al. (2013).
	Activation- Deactivation Adjective Checklist	Fuegen and Breitenbecher (2018).
	Positive and Negative Affect Schedule	Bielinis et al. (2019); Bielinis et al. (2021); Bielinis, Omelan et al. (2018); Bielinis, Takayama et al. (2018); Takayama et al. (2011); Fuegen and Breitenbecher (2018).
	Zuckerman's Inventory of Personal Reactions	Greenwood and Gatersleben (2016);Hassan et al. (2018).
	Overall Happiness Scale	Hartig et al. (2003)
Mental Health	Beck Depression Inventory	Shin and Oh (1996)
	State Trait Anxiety Inventory	Hassan et al.(2018); Lee et al. (2014); Song et al. (2014); Song et al. (2013); Song et al. (2015)
	State Trait Anxiety Inventory- State version	Wang et al. (2016).
Wellbeing	Basler Well-Being Questionnaire	Kelz et al. (2015)
	Recovery-Stress Questionnaire	Kelz et al. (2015)

Outcome	Tool or measurement	Studies
Perceived Restoration	Restorative Outcome Scale	Bielinis et al. (2019); Bielinis et al. (2021); Bielinis, Omelan et al. (2018); Bielinis, Takayama et al. (2018); Takayama et al. (2011);
	Perceived Restorativeness Scale	Wang et al. (2016); Kelz et al. (2015)
Stress	Heart Rate (Beats per minute)	Greenwood and Gatersleben (2016); Lee et al. (2011); Lee et al. (2014); Park et al. (2010); Song et al. (2014); Song et al. (2015); Song et al . (2013); Tsunetsugu et al. (2013)
	Electrocardiogram (R-R intervals)	Wang et al. (2016)
	Heart Rate Variability (low-frequency and high-frequency band variance, R-R intervals)	Lee et al. (2011); Lee et al. (2014); Park et al. (2010); Song et al. (2014); Song et al. (2013).
	Blood Pressure (Systolic and Diastolic)	Greenwood and Gatersleben (2016); Hartig et al. (2003); Lee et al. (2011); Tsunetsugu et al. (2013); Hassan et al.(2018); Park et al. (2010); Lee et al. (2014); Kelz et al. (2015)
	Salivary Cortisol	Lee et al. (2011); Park et al. (2010)
	Serum Cortisol and Testosterone	Mao et al. (2012)
	Electroencephalogram (High Alpha, High Beta)	Hassan et al. (2018)
	Skin Conductance Response	Wang et al. (2016)

Outcome	Tool or measurement	Studies
Energy	Subjective Vitality Scale	Bielinis et al. (2019); Bielinis et al. (2021); Bielinis, Omelan et al. (2018); Bielinis, Takayama et al. (2018); Takayama et al. (2011)
	Activation-Deactivation Adjective Checklist (energy and tiredness)	Fuegen and Breitenbecher (2018)
	Stress-refresh Feeling Test	Tsunetsugu et al. (2013); Lee et al. (2011); Lee et al. (2014)
Cognitive Functioning	Necker Cube Pattern Test	Greenwood and Gatersleben (2016);Hartig et al. (2003)
	Digit Span Backwards	Fuegen and Breitenbecher (2018); Wang et al. (2016)
	Attention Network Task	Kelz et al. (2015)
	Search and Memory Task	Hartig et al. (2003)
	The Symbol Digit Modalities Test	Kelz et al. (2015)
Resilience	Resilience Scale - Shortened Version	Scarf et al. (2017); Scarf et al. (2016); Hayhurst et al. (2015)
Self-concept	Rosenberg Self-esteem Scale	McAnally et al. (2018); Wood et al. (2013).
	Single Item Self-esteem Scale	Hunter et al. (2013)
	Self-Description Questionnaire III	Hayhurst et al. (2015)
	General Self-efficacy Scale	Hayhurst et al. (2015)
Pro-social Behaviour	Strengths and Difficulties Questionnaire	McAnally et al. (2018)
	Empathic Concern Sub-scale	McAnally et al. (2018

Table S2. Summary of Papers by Outcomes

Outcome		Weak		Moderate		Strong	
		NS	PS	NS	PS	NS	PS
Mood and Affect	Negative Mood		Bielinis et al. (2021); Lee et al. (2011); Lee et al. (2014); Mao et al. (2012); Park et al. (2011); Park et al. (2010); Song et al. (2014); Song et al. (2015);Song et al.(2013)	Tsunetsugu et al. (2013); Wood et al. (2013)	Bielinis et al. (2019); Bielinis, Takayama et al. (2018); Takayama et al. (2011)		Bielinis, Omelan et al. (2018)
	Positive Mood		Bielinis et al. (2021); Lee et al. (2011); Lee et al. (2014); Mao et al. (2012); Park et al. (2011); Park et al. (2010); Song et al. (2014); Song et al. (2015); Song et al . (2013)	Tsunetsugu et al. (2013); Bielinis et al. (2019).	Bielinis, Takayama et al. (2018); Takayama et al. (2011)		Bielinis, Omelan et al. (2018)

**Outcome**		**Weak**	**Moderate**	**Strong**			
		**NS**	**PS**	**NS**	**PS**	**NS**	**PS**
	Negative Affect	Bielinis et al. (2021)		Bielinis et al. (2019); Fuegen and Breitenbecher (2018)	Takayama et al. (2011)	Bielinis, Omelan et al. (2018)	
Mental Health	Anxiety		Hassan et al. (2018); Lee et al. (2014); Song et al. (2014); Song et al. (2015)		Wang et al. (2016)		
	Depression		Shin and Oh (1996)				
Wellbeing			McAnally et al. (2018)		Kelz et al. (2015)		
Restoration	Perceived Restoration		Bielinis et al. (2021)		Bielinis et al. (2019); Bielinis, Takayama et al. (2018); Takayama et al. (2011); Wang et al. (2016); Kelz et al. (2015)		Bielinis, Omelan et al. (2018)
Cognitive Functioning	Attention		Greenwood and Gatersleben (2016)	Fuegen and Breitenbecher (2018)	Hartig et al. (2003); Wang et al. (2016)		
	Executive Functioning			Kelz et al. (2015)			

Outcome		Weak	Moderate	Strong			
		NS	PS	NS	PS	NS	PS
	Electrocardiogram				Wang et al. (2016)		
	Heart Rate Variability		Lee et al. (2011); Lee et al. (2014); Park et al. (2010); Song et al. (2014); Song et al. (2015) Song et al . (2013)				
	Blood Pressure	Greenwood and Gatersleben (2016); Lee et al. (2011)	Hassan et al.(2018); Park et al. (2010); Lee et al. (2014)		Tsunetsugu et al. (2013); Kelz et al. (2015); Hartig et al (2003)		
	Cortisol		Lee et al. (2011); Mao et al. (2012); Park et al. (2010)				
	Electroencephalogram		Hassan et al.(2018)				
	Skin Conductance				Wang et al. (2016)		
Energy	Vitality		Bielinis et al. (2021)		Bielinis et al. (2019); Bielinis, Takayama et al. (2018); Takayama et al. (2011)		Bielinis, Omelan et al. (2018)

Outcome		Weak	Moderate	Strong			
		**NS**	**PS**	**NS**	**PS**	**NS**	**PS**
	Energy				Fuegen and Breitenbecher (2018)		
	Refreshed		Lee et al. (2014); Lee et al. (2011)		Tsunetsugu et al. (2013)		
Resilience			Hayhurst et al. (2015)		Scarf et al. (2017); Scarf et al. (2016)		
Self-concept	Self-esteem		McAnally et al. (2018); Hunter et al. (2013); Hayhurst et al. (2015)	Wood et al. (2013)			
	Self-efficacy		Hayhurst et al. (2015)				
Pro-social behaviour	Pro-social behaviour	McAnally et al. (2018)					
	Strengths and difficulties	McAnally et al. (2018)					
	Empathy	McAnally et al. (2018)					

Table S3. Component Ratings for EPHPP Quality Appraisal Assessment

Authors (Year)	EPHPP Criteria for Quantitative Studies						
	Selection Bias	Study Design	Confounders	Blinding	Data Collection Method	Withdrawals and Dropouts	Overall Rating
Bielinis et al. (2019)	Moderate	Strong	Weak	Moderate	Strong	Strong	Moderate
Greenwood and Gatersleben (2016)	Moderate	Strong	Weak	Weak	Moderate	Not Applicable	Weak
Hassan et al.(2018)	Moderate	Strong	Weak	Weak	Weak	Not Applicable	Weak
Bielinis et al. (2021)	Moderate	Strong	Weak	Weak	Strong	Not Applicable	Weak
Bielinis et al. (2018a)	Moderate	Strong	Strong	Moderate	Strong	Weak	Moderate
Bielinis et al. (2018b)	Moderate	Strong	Weak	Moderate	Strong	Not Applicable	Moderate
Hartig et al. (2003)	Moderate	Strong	Strong	Weak	Moderate	Not Applicable	Moderate
Lee et al. (2011)	Moderate	Strong	Moderate	Weak	Weak	Not Applicable	Weak
Lee et al. (2014)	Strong	Strong	Moderate	Weak	Weak	Not Applicable	Weak
Mao et al. (2012)	Moderate	Strong	Moderate	Weak	Weak	Not Applicable	Weak
Park et al. (2011)	Moderate	Strong	Weak	Weak	Strong	Not Applicable	Weak
Park et al. (2010)	Moderate	Strong	Weak	Weak	Weak	Not Applicable	Weak
Song et al. (2014)	Moderate	Weak	Strong	Weak	Weak	Not Applicable	Weak
Song et al. (2015)	Moderate	Weak	Strong	Weak	Weak	Not Applicable	Weak
Song et al . (2013)	Moderate	Weak	Strong	Weak	Strong	Not Applicable	Weak
Takayama et al. (2011)	Moderate	Strong	Strong	Weak	Strong	Not Applicable	Moderate
Tsunetsugu et al. (2013)	Moderate	Strong	Moderate	Weak	Strong	Not Applicable	Moderate
McAnally et al. (2018)	Moderate	Moderate	Strong	Weak	Strong	Weak	Weak
Fuegen and Breitenbecher (2018)	Moderate	Strong	Strong	Weak	Strong	Not Applicable	Moderate
Shin and Oh (1996)	Moderate	Moderate	Strong	Weak	Strong	Weak	Weak

**Authors (Year)**	**EPHPP Criteria for Quantitative Studies Selection Bias**	**Study Design**	**Confounders**	**Blinding**	**Data Collection Method**	**Withdrawals and Dropouts**	**Overall Rating**
Wood et al. (2013)	Strong	Strong	Strong	Weak	Strong	Strong	Moderate
Kelz et al. (2015)	Strong	Moderate	Strong	Weak	Strong	Moderate	Moderate
Scarf et al. (2017)	Moderate	Weak	Strong	Strong	Strong	Weak	Weak
Scarf et al. (2016)	Moderate	Moderate	Moderate	Moderate	Strong	Weak	Moderate
Hayhurst et al. (2015)	Moderate	Moderate	Strong	Weak	Strong	Weak	Weak
Hunter et al. (2013)	Moderate	Moderate	Strong	Weak	Moderate	Weak	Weak
